# 4224 Development of a Patient Registry and Biospecimen Repository to Identify Biomarkers in Nontuberculous Mycobacteria Lung Disease

**DOI:** 10.1017/cts.2020.382

**Published:** 2020-07-29

**Authors:** Bryan Garcia, Abigail Grady, Lilian Christon, Patrick Flume, Susan Dorman

**Affiliations:** 1 Medical University of South Carolina; 2 MUSC

## Abstract

OBJECTIVES/GOALS: Non-tuberculous mycobacteria (NTM) is an emerging infection in the United States. Therapeutic development is impaired in NTM due to lack of biomarkers associated with disease activity and treatment response. To address this, we established a cross-sectional patient registry and biorepository from NTM patients. METHODS/STUDY POPULATION: Beginning August 2019 patients were recruited in a cross-sectional format from the bronchiectasis and NTM clinics at our institution. All patients provided at least one sputum sample in the six months prior to inclusion. Clinical and epidemiologically relevant data was obtained, patient reported outcome measures including the SGRQ were provided, and blood specimens were processed and preserved. Patients were grouped based on clinical phenotype and descriptive statistics were reported as means and standard deviations. Serum inflammatory profiles will be analyzed using standard Luminex assays. RESULTS/ANTICIPATED RESULTS: 72 patients with prior NTM isolation from sputum have been recruited including 28 patients that do not meet ATS guidelines for NTM treatment (colonized), 29 patients are currently receiving treatment, and 15 patients that have a history of completing therapy. Among all NTM patients, the mean age was 59.5 ±17.6 years and 80.8% were female. The mean FEV_1_ percent predicted among these patients was 68.0 ±21.5% and mean BMI was 22.35 ±4.1. The most common mycobacterial species isolated was Mycobacterium Avium Complex (47%). The mean SGRQ total score was 40.2 ±20.3 among all NTM patients. We plan to perform standard Luminex assays to identify inflammatory profiles associated with disease phenotype. DISCUSSION/SIGNIFICANCE OF IMPACT: We developed a cross sectional cohort of NTM patients including a registry of clinically relevant data for phenotyping and an accompanying biospecimen repository. Our long-term goal is to identify biomarkers indicative of disease activity and treatment response using these components.

